# Clustering procedures for the optimal selection of data sets from multiple crystals in macromolecular crystallography

**DOI:** 10.1107/S0907444913012274

**Published:** 2013-07-20

**Authors:** James Foadi, Pierre Aller, Yilmaz Alguel, Alex Cameron, Danny Axford, Robin L. Owen, Wes Armour, David G. Waterman, So Iwata, Gwyndaf Evans

**Affiliations:** a Membrane Protein Laboratory, Diamond Light Source, Harwell Science and Innovation Campus, Didcot, Oxfordshire OX11 0DE, England; b Division of Molecular Biosciences, Imperial College, London SW7 2AZ, England; c Diamond Light Source, Harwell Science and Innovation Campus, Didcot, Oxfordshire OX11 0DE, England; d Oxford e-Research Centre (OeRC), Keble Road, Oxford OX1 3QG, England; e CCP4, Research Complex at Harwell (RCaH), Harwell Science and Innovation Campus, Didcot, Oxfordshire OX11 0FA, England

**Keywords:** clustering, multiple crystals, *BLEND*, scaling, merging, multi-crystal data sets

## Abstract

The availability of intense microbeam macromolecular crystallography beamlines at third-generation synchrotron sources has enabled data collection and structure solution from microcrystals of <10 µm in size. The increased likelihood of severe radiation damage where microcrystals or particularly sensitive crystals are used forces crystallographers to acquire large numbers of data sets from many crystals of the same protein structure. The associated analysis and merging of multi-crystal data is currently a manual and time-consuming step. Here, a computer program, *BLEND*, that has been written to assist with and automate many of the steps in this process is described. It is demonstrated how *BLEND* has successfully been used in the solution of a novel membrane protein.

## Abstract 2

A systematic approach to the scaling and merging of data from multiple crystals in macromolecular crystallography is introduced and explained.

## 1. Introduction

The formation of good-sized and well diffracting crystals from large and/or insoluble proteins remains a significant challenge in macromolecular crystallography (MX). Progress has certainly been made in recent times (Fry *et al.*, 1999 ▶; Caffrey, 2003 ▶; Carpenter *et al.*, 2008 ▶), but obtaining good-quality crystals of viruses, large molecular complexes or membrane proteins is still challenging today.

Even in cases in which small crystals are obtained, very few data can be collected before they receive a damaging radiation dose. Consequently, crystallographers are often unable to collect complete data sets from small crystals of a few micrometres in size (Holton & Frankel, 2010 ▶).

A possible solution to these limitations is to merge data sets from many crystals into a single data set. This practice is not new, since for isomorphous replacement or multi-wavelength anomalous diffraction data necessarily have to be combined from multiple sets. This has also been a standard approach in virus crystallography, where room-temperature data collection from highly sensitive samples necessarily implies the merging of data from many crystals (Wang *et al.*, 2012 ▶). The need to deal with multiple data sets is nowadays becoming more and more common, mainly because of greater accessibility to microfocus beamlines at synchrotrons around the world. A micrometre-sized beam can be effectively used for data collection from difficult crystals in a number of ways:

(i) a large crystal can be hit by the microbeam at different locations during rotation, thereby continually introducing unexposed volumes of the crystal to X-rays and reducing the impact of radiation damage on the resulting data;
(ii) small crystals that diffract very weakly on standard beamlines can instead produce respectable diffraction patterns using intense microbeams; and
(iii) small-sized crystals can produce weak but sufficiently interpretable diffraction patterns when they are exposed directly inside crystallization plates (Watanabe *et al.*, 2002 ▶; Ng *et al.*, 2008 ▶; le Maire *et al.*, 2011 ▶; Soliman *et al.*, 2011 ▶; Axford *et al.*, 2012 ▶). The obvious advantage of this *in situ* technique is that the whole time-consuming procedures behind loop fishing and crystal cooling can be avoided, thus making the method fast and amenable to automation.

If the number of recorded spots and their signal-to-noise ratio are sufficiently high to allow indexing, integration does not usually pose any significant problem for data sets yielded by multiple crystals. However, the merging of such potentially diverse and very partial data sets can be a challenge.

Isomorphous crystals will give rise to data whose merging exhibits little error. In contrast, merging data from non-isomorphous crystals yields data sets with bad statistics, and if the degree of non-isomorphism is very high, merging may even fail. Therefore, one approach to building a complete data set from partial data sets from multiple crystals is to select and group together those crystals that have a higher likelihood of merging well together.

Besides the obvious advantage of providing complete native data sets, data collection from multiple crystals has also shown potential to reduce systematic errors and to strengthen the anomalous signal in the SAD and MAD phasing techniques (Liu *et al.*, 2011 ▶). A remarkable *tour de force* in data integration and scaling from multiple microcrystals was recently performed by Hanson *et al.* (2012 ▶). The method adopted by Hanson *et al.* (2012 ▶) is applicable to cases in which the data are strongly affected by radiation damage and the only way to obtain a fairly complete data set to a reasonable resolution is through data collection from very many crystals. The procedure consists of several steps and makes use of recycling between integrated and scaled data, with the progressive inclusion of individual observations, based on the value of the corresponding merging statistics. Owing to the need for frequent human intervention to judge the removal or acceptance of groups of observations, this approach is more akin to a protocol than to an automated or semi-automated procedure.

This paper describes a method to assist in the selection of optimal groups of data sets from multiple crystals prior to scaling and merging. Cluster analysis forms the central part of this approach and is implemented in a computer program called *BLEND*. The following sections introduce the statistical descriptors used to characterize data sets and the clustering method adopted, their implementation in *BLEND* and several practical examples.

## 2. Theory and methods

**Figure 1:**
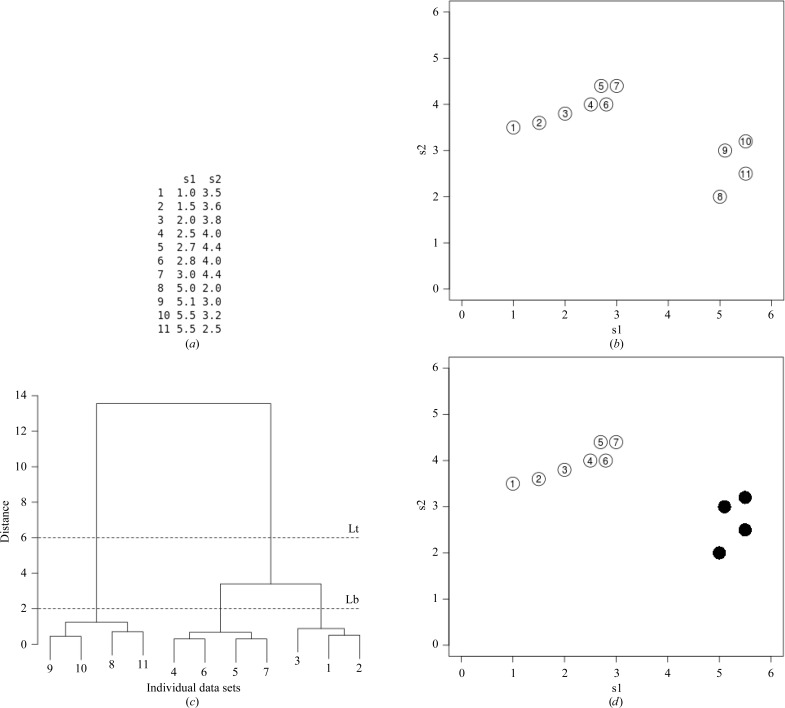
Main steps involved in the type of cluster analysis used in this paper. (*a*) 11 data sets are characterized with 22 numerical values taken by two statistical descriptors. (*b*) These data sets can be represented in a two-dimensional space (a plane) as there are only two descriptors. (*c*) Hierarchical cluster analysis carried out using Ward linkage gives rise to this dendrogram. Data sets and clusters merge into progressively larger groups as the cluster height considered is increased. (*d*) Specific clusters corresponding to merging nodes can be isolated using one or two height levels. Only data sets 1–7 are selected in this example because only one merging node is included between levels Lt and Lb.

*BLEND* carries out an analysis of multiple data sets at the pre-scaling stage, working exclusively with integrated but unscaled and unmerged intensities. The main objective of the procedure is to partition multiple data sets into homogeneous groups whose merging statistics have a tendency to show optimal values. A visual rendering of the procedure is depicted in Fig. 1 ▶, where partitioning and selection are explained using a simulated example with 11 data sets. Multivariate statistics offers several techniques to accomplish such partitioning. Cluster analysis is among the most frequently used, especially at an exploratory stage where not much is known about the probability distribution underlying the objects being investigated. The scenario faced by the researcher merging multiple integrated data sets is very much the typical scenario found in exploratory data analysis, where groups are contemplated in an otherwise unshaped set. A good and thorough introduction to cluster analysis is given by Everitt *et al.* (2011 ▶).

### 2.1. Main elements of the clustering procedure

In our procedure for cluster analysis, each wedge (a contiguous series of diffraction images) of data is treated as a geometric point in multidimensional space. Points which are in close proximity are deemed to belong to the same group. Grouping is performed in a hierarchical way, in which small nuclei of close points are progressively enlarged to include more and more elements until the whole set of data wedges is engulfed in a final all-inclusive cluster. The procedure and the main concepts are now introduced.

#### 2.1.1. Statistical description of data sets and clusters

Individual data objects are characterized through *statistical descriptors*. These are numerical quantities associated with selected features of each object, whose aim is to describe quantitatively specific parts of data variability. If *m* statistical descriptors are associated with each object, then all data will be represented as points in an *m*-dimensional orthonormal space. Distances between all pairs of points in *m*-dimensional space are computed and the two closest objects are merged to form the first cluster. Although several definitions of distance are possible in cluster analysis, Cartesian distance, as adopted here, is appropriate and most common when the underlying statistical descriptors show continuous variability across real numbers.

#### 2.1.2. Cluster-linkage method

After the formation of the first cluster of two elements, the next two closest points are merged to form the second cluster. In measuring all distances during this second pass one includes the possibility of measuring the distance between an individual point and a cluster. In *BLEND* the proximity of a point to a cluster (or between two clusters) is defined as the distance between the point and the cluster centroid(s).

The annexation of points or clusters to form larger clusters is known as the *linkage method*. None of the existing linkage methods are known to perform universally well and the appropriate method is selected according to the specific application. In the context of obtaining good merged data sets from multiple data wedges it is desirable to form groups with features that are not too dissimilar among elements within a cluster. For this reason it is advisable to use linkage methods that use centroids. Among these methods, algorithms to implement centroid and Ward linkage are very popular and are readily available. In the centroid linkage the proximity between two clusters is defined as the distance between the centroids of each cluster. In the Ward linkage the proximity is given by the increase in distance variance obtained when the two separate clusters are joined into a single cluster (see, for instance, p. 77 of Everitt *et al.*, 2011 ▶). Both types of linkage method tend to produce compact clusters, but the centroid method is subject to the undesirable possibility of *inversion*. This means that two clusters merging at a given step can happen to be more similar than clusters that merged in a previous step (see, for instance, Tan *et al.*, 2006 ▶). In the framework that we have developed, in which data sets merge in order of decreasing proximities, this is an unwanted feature. For this reason, and on the basis of empirical tests, we have decided to work with the Ward linkage.

#### 2.1.3. Dendrograms

The overall picture of hierarchical clustering can be effectively illustrated by the dendrogram, a kind of inverted tree whose leaves symbolize individual data sets and whose branches progressively merge into each other and eventually into an all-encompassing trunk. A numeric vertical scale, or cluster height, is shown at the side of the tree to give a quantitative measure of the distances at which smaller branches merge into larger branches. Through the selection of one or two values on this vertical scale, a certain number of clusters can be highlighted. In Fig. 1 ▶ the whole process is illustrated with a simulated example involving 11 data sets and two statistical descriptors. A review of hierarchical clustering is given by Jain *et al.* (1999 ▶).

### 2.2. Statistical descriptors

The effectiveness of cluster analysis relies to a great extent on how well the statistical descriptors chosen for data characterization are able to capture their essential features. Differences in the numerical values of such quantities should correlate to physical differences among the associated data sets and structures. It is not clear how to select descriptors when dealing with data immediately after integration, as these have not yet been scaled to produce structure factors. Furthermore, issues concerning indexing and choice of the correct space group are still present at the pre-scaling stage. If integrated intensities are selected to build descriptors, then their averages in resolution shells, rather than individual values, will have to be used. Other quantities available after data integration are unit-cell parameters and mosaicity. The last can be a measure of crystal packing, but it is very often plagued by correlations with other experimental quantities during post-refinement. Unit-cell parameters can also be affected by such correlations, but to a lesser extent. Indeed, we have found mosaicity not to be an effective and reliable descriptor, and have not used it. On the other hand, unit-cell parameters have shown a remarkable ability to signal similarities and differences among data sets. For this reason, unit-cell parameters are currently the only descriptors used in *BLEND*.

If no significant variation in unit-cell parameters occurs across multiple data sets, then it can be expected that each parameter will be distributed around a single average. When different crystals (or different data sets from one crystal) exhibit non-isomorphism, the population of unit cells can break up into groups with unit-cell parameters distributed around two or more averages.

This can be induced by a change in molecular packing, by a genuine variation in parts of one or more chains composing the structure, by an alteration in the amount of bulk solvent or by a combination of these factors. Indeed, changes in unit-cell parameters caused by structural variations were observed during the first crystallographic studies of macromolecules (Perutz, 1946 ▶), as the isomorphous replacement method was then the only available technique to solve protein structures. More recently, dehydration techniques have enabled researchers to explore crystal packing and structural change in a systematic way (Pickford *et al.*, 1993 ▶; Esnouf *et al.*, 1998 ▶; Bowler *et al.*, 2006 ▶; Russi *et al.*, 2011 ▶).

It is important to stress that in order to be effective in the determination of clusters of data sets, unit-cell parameters have to be filtered through principal component analysis and statistical standardization (where data are rescaled so as to have a mean equal to 0 and a standard deviation equal to 1) prior to cluster analysis. This has the beneficial effect of removing undesired correlations among descriptors and of placing all of them on the same numerical scale.

The kind of standardization just described allows data to be prepared for cluster analysis in an appropriate way, because all descriptors are given equal numerical weight. However, this operation also has the undesirable effect of removing the ability to visually detect non-isomorphism between different groups of data sets. The cluster height appearing in the dendrograms presented in this work has no immediate connection with the variation of unit-cell parameters in absolute terms. Thus, for instance, and as will be described later in the text (see §§[Sec sec3.3]3.3 and [Sec sec3.4]3.4), the nine data sets of the ultralente structure can be divided into two groups which present a good degree of non-isomorphism, while the 11 data sets for *in situ* lysozyme do not show any major non-isomorphism. Still, both dendrograms display a clear separation into two distinct groups, and the cluster height does not make it clear that this separation reflects isomorphism issues. It is therefore useful to use some quantitative measure of unit-cell parameter variation in addition to cluster analysis in order to assess the real variation of unit cells, as this is not detectable in the dendrogram. Among the many parameters that can be imagined, we have found that a quantity describing a maximum linear variation that also takes into account unit-cell angles is best suited for the characterization. Such a quantity, named linear cell variation (LCV), measures the maximum linear increase or decrease of the diagonals on the three independent cell faces; details are given in Appendix *A* [App appa]. LCV values for the five test cases treated in §[Sec sec3]3 are shown in Table 1 ▶.

### 2.3. The program structure

*BLEND* can be executed in two modes: analysis and synthesis. Program execution is regulated by keywords included in an ASCII file. Input data are unmerged reflection files in MTZ or XDS format. The final output is a number of merged reflection files in MTZ format.

Initially, *BLEND* is run in analysis mode (option -a). The input is a list of data sets to be processed or the path to the directory containing the data. The program checks each data set in turn, making sure that it is properly formatted and includes integrated data taken in a continuous sweep. Data sets failing this check are discarded and an updated file list is stored as a new ASCII file.

Next, it applies the unit-cell parameter filtering of §[Sec sec2.2]2.2 and performs the cluster analysis (§[Sec sec2.1]2.1). The output of the analysis mode is a cluster dendrogram and an associated ASCII file providing details of the clusters. Information obtained during execution in analysis mode is also dumped to a binary file, ready to be read by the program when running in synthesis mode. Users should both look at the dendrogram and read through its ASCII counterpart to decide which clusters of data sets are worth further investigation.

Once one or more clusters have been singled out, *BLEND* is run in synthesis mode (option -s). It will suffice to provide one or two numerical levels corresponding to cluster-height values (see §[Sec sec2.1]2.1 and Figure 1 ▶). The program will accordingly scale and merge together all data sets at each node included in the specific dendrogram region. Scaling and merging are carried out using the *CCP*4 programs *POINTLESS* and *AIMLESS* (Evans, 2006 ▶).

Execution in synthesis mode can be repeated with different numerical level values as many times as required. Specific keywords for either *POINTLESS* or *AIMLESS* can be added in *BLEND* keywords files so that execution in synthesis mode can be customized. *BLEND* can also be executed in combination mode (option -c) when the user wishes to combine data sets outside an existing cluster. This mode is particularly useful when reference data sets need to be included to stabilize the scaling of many incomplete ones, a practice that is quite often followed by protein crystallographers. Unmerged files produced by *POINTLESS* containing the most likely space group, or one selected by the user, are also stored, ready to be used for prospective and individual scaling jobs, separately from *BLEND*.

*BLEND* requires only minimal and simple intervention from the user, but also permits both analysis and synthesis modes to be tailored to specific needs should the user desire.

## 3. Test cases, results and discussion

Five test cases have been selected to demonstrate the use and usefulness of *BLEND*.

In each test case, the resolution limits were selected to be the same for all clusters. This was performed in a conservative fashion so as to have 〈*I*/σ(*I*)〉 ≥ 2 for all individual data sets. Such limits can easily be changed by the user through the keywords file. Details of data collection for all five test cases are included as Supplementary Material^1^. All data were integrated using either *XDS* (Kabsch, 1993 ▶) or *MOSFLM* (Leslie, 1992 ▶).

A warning should be issued for dendrogram inspection. As explained in §[Sec sec2.2]2.2, cluster analysis has a tendency to create partitions out of any group of objects, even when such partitions do not naturally exist. Therefore, the splitting of dendrograms into branches does not necessarily correspond to major physical differences between data sets. As also explained in §[Sec sec2.2]2.2, a complementary overall quantity, the LCV, will give us a quantitative feeling of the degree of isomorphism existing among each group of data sets. LCV values for the five test cases explored here are included in Table 1 ▶. The case showing the highest variability is that of ultralente insulin, while *in situ* lysozyme is the case with the lowest cell variability. These two cases were selected for thorough structural investigations of non-isomorphism (see §§[Sec sec3.3]3.3 and [Sec sec3.4]3.4).

A few words on the traditional merging statistics used to measure data quality also need to be included. Until recently, these statistics were drawn from data obtained as single complete sweeps from a single crystal. The appreciation of specific numeric values as indicators of good data quality has evolved during several years of steady, and often nonproblematic, collection from such crystals. With the recent advent of techniques from multiple crystals and with the diffusion of problematic data sets (from membrane proteins, viruses and complexes) this appreciation will have to be widened to allow the inclusion of data that would otherwise be discarded. The determination and numeric values of merging statistics depending on geometric factors, such as completeness or multiplicity, is not affected by the multiple-crystal scenario. Other statistics, however, such as *R*_merge_, *R*_meas_, *R*_p.i.m._ *etc.* will in general tend to be higher, often assuming values that would be deemed unacceptable if the same data had been collected from a single crystal. Furthermore, the scaling process itself normally finds problems when dealing with multiple data sets, even when non-isomorphism is not an issue. In our experience the estimation of errors for scaled intensity has proved to be particularly problematic, even in those cases where the data sets came from the same crystal. This fact obviously has repercussions on the determination of one of the traditional quality indicators, 〈*I*/σ(*I*)〉. In the five test cases reported here we have used *AIMLESS* for all scaling jobs. The algorithm used in this program for the automatic determination of the SDFAC/SDAAD parameters is not stable when applied to multiple incomplete data sets (Evans, 2013 ▶). An effort was made to manually adjust these parameters as best as possible and the best efforts have been included in all tables presenting merging statistics. While we acknowledge the importance of the estimation of 〈*I*/σ(*I*)〉 as an effective way to judge data quality, it is also important to stress that its evaluation has had no influence on the other results and findings presented in this paper.

### 3.1. Cryocooled thaumatin: space group *P*4_1_2_1_2

14 data sets were collected from 12 crystals (three data sets were collected at different positions from a large crystal) on beamline I02 (using an ADSC Q315r CCD detector) and beamline I24 (using a Dectris PILATUS 6M detector) at Diamond Light Source, UK. Crystal sizes varied from 30 × 30 to 500 × 500 µm. Angular rotation ranges varied from 45° to 360° sweeps. Rotation steps were also very different from data set to data set (0.15° to 0.5° per image). All data sets were truncated to include only the first 40 images for the purposes of testing *BLEND* in order to avoid unnecessary lengthy runs while testing and to mimic what might be more commonly happening when data sets from smaller multiple crystals are collected.

**Figure 2:**
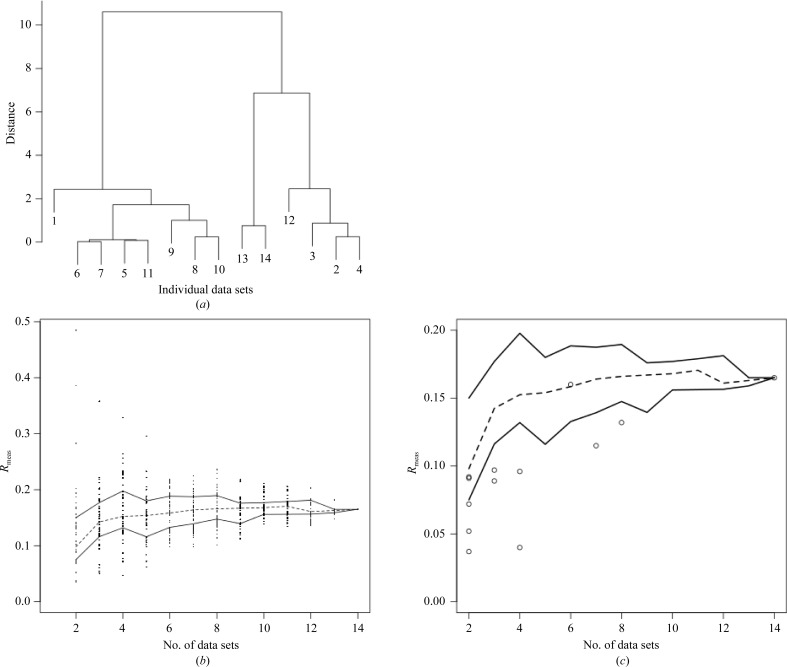
(*a*) Dendrogram for cluster analysis of the 14 cryocooled thaumatin data sets introduced in §[Sec sec3.1]3.1. (*b*) *R*_meas_ for random combinations of the 14 data sets introduced in §[Sec sec3.1]3.1. Calculations for groups of two, three, four all the way up to 14 data sets are shown. The broken line runs through the medians for all groups, while the full lines include the inter-quartile range, *i.e.* all dots falling below the lower line and all dots falling above the upper line represent 50% of all values. Optimally selected groups of data sets could be considered as those having *R*_meas_ below the lower full line; these are included among the 25% of best-performing groups. (*c*) The broken and full lines in this plot are a replica of those in (*b*). The empty circles correspond to values of *R*_meas_ for all merged data sets found in the dendrogram in (*a*). Ten out of 13 of them fall under the lower inter-quartile range line. We know that only data sets performing among the top 25% fall in this region. Thus, the selective power provided by cluster analysis is quite evident.

The analysis mode of *BLEND* on these 14 data sets produced the dendrogram shown in Fig. 2 ▶(*a*).

There are 91 ways to combine two different data sets taken from the 14 available, 364 ways to combine three different data sets, 1001 ways to combine four different data sets and so on. In order to quantify how well different groups of data sets merged together, and in light of the very large number of possible merging combinations, a random selection of 50 combinations from all groups of size 2, 3, 4 or more were merged. Given that there are only 14 ways to form different combinations of 13 data sets and only one combination of 14 data sets, all of these combinations were included in the test. It still took a few hours on a standard laptop PC to run *POINTLESS* and *AIMLESS* of all sampled groups (there were (11 × 50) + 14 + 1 = 565 runs of *POINTLESS* and *AIMLESS*). The highest resolution chosen for all scaling jobs was 1.95 Å.

The results are presented in Fig. 2 ▶(*b*) and show the spread of *R*_meas_ values for all combinations. *AIMLESS* was run using default options only, resulting in 41 failed jobs, so only 524 points appear in the plot. Data overlap between sets and completeness can be low for data sets with small group sizes and would explain why the *R*_meas_ spread is larger for these cases. At the same time, however, for data sets showing a higher degree of isomorphism the *R*_meas_ would tend to be lower than for combinations with a higher number of data sets. This has been explained by Diederichs & Karplus (1997 ▶) and Weiss (2001 ▶) as the statistical tendency of *R*_meas_ to remain stationary or to increase slightly when the number of unmerged reflections is increased. A slight increase generally indicates the addition of data with good isomorphism, while a larger increase is normally taken to indicate the addition of data which are in part non-isomorphous.

The best merged data sets as indicated by *BLEND*, corresponding to the merging nodes of the dendrogram in Fig. 2 ▶(*a*), are plotted in Fig. 2 ▶(*c*) together with the median, upper and lower inter-quartile lines of Fig. 2 ▶(*b*).

In this example ten out of 13 of the *BLEND* data sets lie below the lower interquartile line, illustrating how it is able to select groups of data sets for merging that have a high likelihood of showing optimal statistics. (See Table 2 ▶ for a summary of the merging statistics.) *BLEND* therefore presents a very quick route to obtaining optimal combinations of data wedges for further downstream analysis.

### 3.2. Cryocooled insulin: space group *H*3

These data sets were collected on Diamond MX beamlines I02, I03 and I04. Data were obtained from 14 crystals as short angular sweeps.

**Figure 3:**
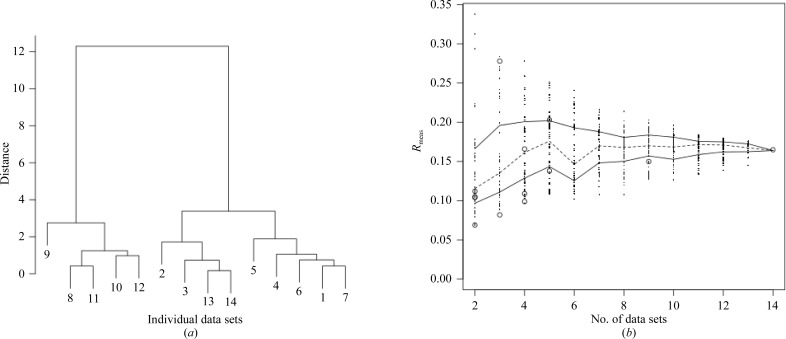
(*a*) Dendrogram for cluster analysis of the 14 cryocooled insulin data sets introduced in §[Sec sec3.2]3.2 and (*b*) the spread of *R*_meas_ values for random combinations of 2, 3, 4, …, 13, 14 data sets from the same group of data. The broken line joins the medians for all cases. Full lines join the inter-quartile range points for all cases. The empty circles represent *R*_meas_ for all merged data sets found in the dendrogram. Seven out of 13 of them fall under the lower inter-quartile range line. However, 11 out of 13 fall below the median line. Although the selections suggested by *BLEND* do not perform as well as in the case of cryocooled thaumatin, still they can be considered to be very good.

The dendrogram from *BLEND* is shown in Fig. 3 ▶(*a*). The same test procedure as used for thaumatin was repeated and the results are presented in Fig. 3 ▶(*b*). The high-resolution cutoff was set to 1.92 Å. In this case only seven out of 13 groups of data sets fall in the lower quartile; however, 11 out of 13 groups still fall below the median line, again illustrating that clustering using unit-cell parameters leads to the selection of better than average merging sets. Numerical values for the selections suggested by *BLEND* are listed in Table 3 ▶.

### 3.3. Cryocooled microcrystals of insulin ultralente: space group *H*3

**Figure 4:**
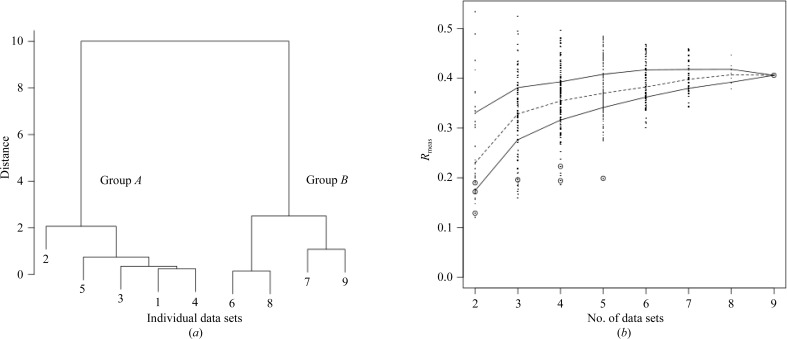
(*a*) Dendrogram for the cluster analysis of ultralente insulin (§[Sec sec3.3]3.3) and (*b*) the spread of *R*_meas_ values for random combinations of data sets from the same group of collected data (right). The open circles represent groups corresponding to nodes in the dendrogram. Data sets 1–5 in the dendrogram form group *A* and data sets 6–9 form group *B*.

Very small (25 × 25 × 5 µm) crystals of ultralente insulin have been used in the treatment of diabetes for a number of years. Because of their small size, the structure of ultralente insulin was only recently solved (Wagner *et al.*, 2009 ▶) using one of many data sets measured from microcrystals. Although the resolution was relatively high, refinement did not perform particularly well (see Table 4 ▶). It was thought that a multiple data-set approach to this problem might yield a better refined data set if isomorphism was conserved to a good degree. Nine data sets collected on beamline X06SA at the Swiss Light Source were used. As there were only nine data sets, all 502 combinations of these were merged and scaled using *POINTLESS* and *SCALA* for comparison to the *BLEND* results. These results, together with the combinations selected by *BLEND*, are shown in Fig. 4 ▶(*b*).

**Figure 5:**
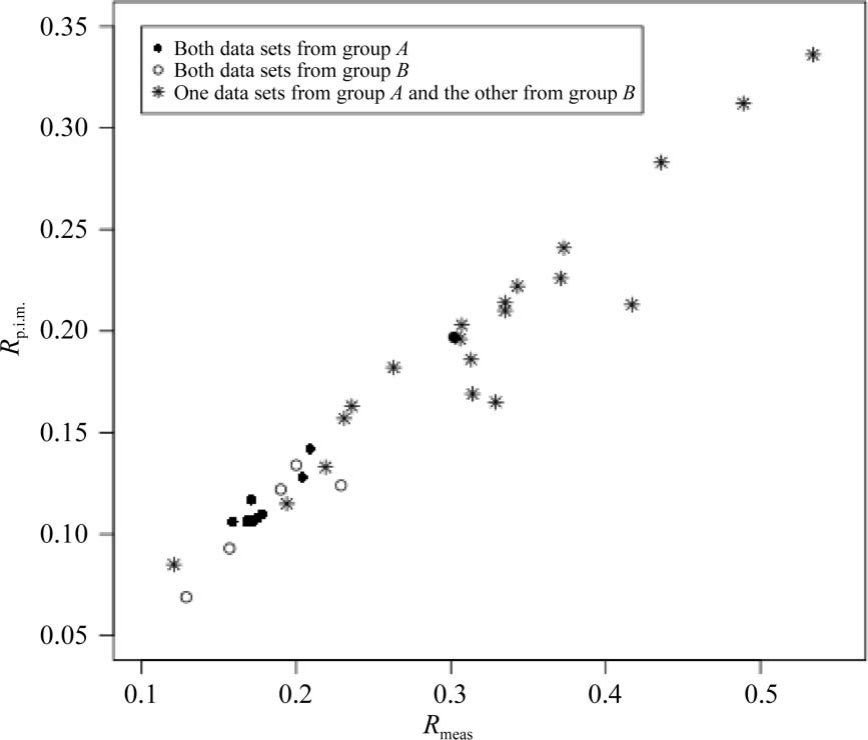
*R*_meas_ *versus* *R*_p.i.m._ values obtained for merging pairs of data sets from the ultralente case described in §[Sec sec3.3]3.3. Full circles relate to pairs within group *A* and empty circles to pairs within group *B*. Stars relate to pairs from different groups. The worst merging statistics are typically seen when the merging is between pairs from different groups.

It can be seen from the dendrogram in Fig. 4 ▶(*a*) that the nine data sets partition into two main groups, denoted here as group *A* (data sets 1–5) and group *B* (data sets 6–9). In order to evaluate whether this grouping reflects real differences between the data sets or is simply a consequence of the clustering procedure, merging statistics, *R*_meas_ and *R*_p.i.m._, between pairs of data sets within and between groups *A* and *B* were calculated and plotted in Fig. 5 ▶. Merging statistics are listed in Table 5 ▶. It is clear that merged pairs within the groups show significantly better statistics. In order to investigate further, structure refinement was performed against group *A* (to 2.1 Å resolution) and group *B* (to 2.0 Å resolution) data and the models were compared. Using an initial model (PDB entry 2vk0; Wagner *et al.*, 2009 ▶) rigid-body refinement was first carried out, followed by alternating cycles of model building with *Coot* (Emsley *et al.*, 2010 ▶) and model refinement with *REFMAC* (Murshudov *et al.*, 2011 ▶). Finally, a few cycles of TLS refinement with *REFMAC* were carried out to improve the model. The same test set as used in Wagner *et al.* (2009 ▶) was used for cross-validation and *R*_free_ calculations. The statistic for the final models, model *A* and model *B*, are shown in Table 6 ▶. Model *A* refined to *R* and *R*_free_ values of 18.5 and 24.8%, respectively, whereas the values for model *B* were 20.9 and 28.0%, respectively. Overall, the two models appear to be similar and the refinement statistics were improved, especially for model *A*, compared with the original structure. The ultralente structure, like other forms of insulin, is a dimer composed of a 21-residue chain (chain A) and a 30-­residue chain (chain B). Two of these dimers are contained within the asymmetric unit, and are described as four separate chains: *A*, *B*, *C* and *D*. The presence of zinc ions facilitates the formation of hexamers following *H*3 symmetry.

Values for the r.m.s. positional difference between C^α^ atoms refined against group *A* and group *B* data for all atoms in the asymmetric unit and the four chains individually vary between 0.33 and 0.48 Å (values are calculated without first superposing the respective molecules). These differences are significant in comparison to the differences in unit-cell parameters between the two structures.

**Figure 6:**
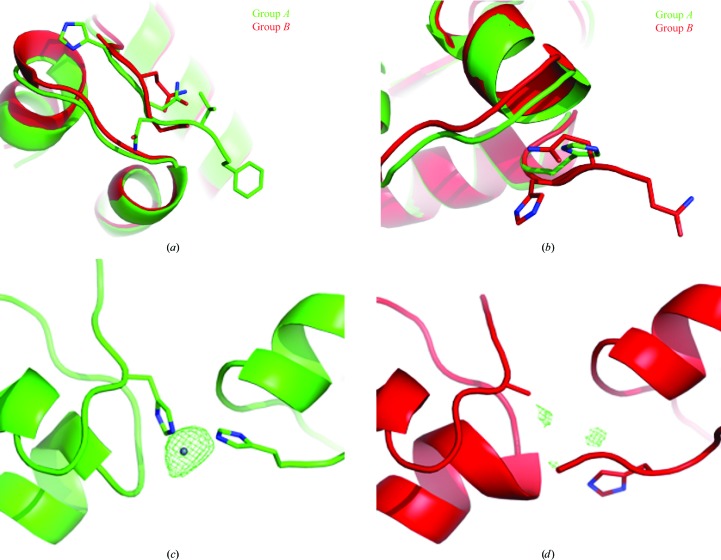
(*a*) Insulin ultralente, where the N-terminus of chain *B* can be modelled for group *A*, but not for group *B*. (*b*) On the other hand, the N-terminus of chain *D* can be modelled for group *B*, but not for group *A*. In chain *D* it is also possible to observe two groups of different rotamers for His5. (*c*, *d*) *F*_o_ − *F*_c_ density contoured at 3σ around one of the zinc ions for (*c*) data in group *A* and (*d*) data in group *B*. While density showing the presence of the ion is very evident in (*c*), it could be concluded quite reasonably that the zinc is missing in (*d*). This is also supported by the different rotamer of His5 in chain *D*; no bonds to a zinc ion could be coordinated by different dimers with the histidine tilted in this way.

This is compatible with some obvious differences at the secondary chain level:

(i) the N-terminus of chain *B* can be modelled in group *A*, but not in group *B* (see Fig. 6 ▶ ▶ *a*),
(ii) the N-terminus of chain *D* can be modelled in group *B*, but not in group *A* (see Fig. 6 ▶ *b*),
(iii) in model *A*, His5 of chain *D* is a different rotamer to that in model *B*, and
(iv) in Fig. 6 ▶ the *F*_o_ − *F*_c_ density map of group *A* shows (at 3σ) one additional Zn ion with respect to group *B*.

Points (iii) and (iv) seem to be responsible to a great extent for the non-isomorphism, because chain *B* is connected to chain *D* of another dimer through a Zn ion only if His5 of chain *D* has the proper rotamer (see Fig. 6 ▶). In this example *BLEND* was able to generate a merged data set from the combination of data from multiple crystals that resulted in an improved structure compared with that refined from a single-crystal data set.

### 3.4. *In situ* room-temperature lysozyme: space group *P*4_3_2_1_2

**Figure 7:**
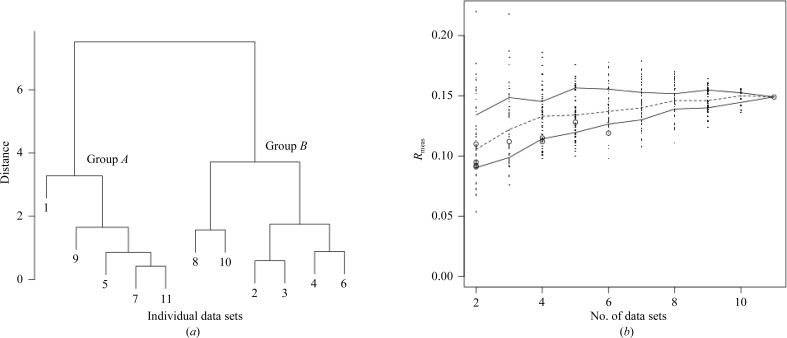
(*a*) Dendrogram derived for *in situ* lysozyme data sets and (*b*) the spread of *R*_meas_ values for random combinations of the same data sets. The two clusters indicated as group *A* and group *B* were singled out to explore the structure isomorphism (see main text).

Test data sets from 12 crystals sitting within crystallization plates were collected on Diamond beamline I04-1. For all of the tests, the known space group *P*4_3_2_1_2 was imposed using the *BLEND* keywords file. Interestingly, only data set 4 yielded a unique and correct space-group determination in *POINTLESS*. Fig. 7 ▶ shows similar results to those obtained previously using 50 random samples to determine the spread of *R*_meas_. Merging statistics are listed in Table 7 ▶. In this case, nine out of ten groups fall below the median and seven of them are very close to the lower inter-quartile range line.

Two major branches appear in the dendrogram in Fig. 7 ▶(*a*), implying the existence of two isomorphous groups. However, cluster analysis has a natural tendency to form small and large clusters out of any collection of objects, even when distinct groups reflect no real macroscopic differences. This also holds true in the dendrograms produced by *BLEND*. The ultimate indicator of isomorphism across crystal components is em­bodied by the merging statistics. A sudden increase in *R*_meas_ (and *R*_p.i.m._) values when two groups of data sets merge to form a larger group can be an indication of non-isomorphism, whereas small variations in *R*_meas_ accompanied by a decrease in *R*_p.i.m._ normally indicate a good degree of isomorphism of the merging groups. In this case the indication is that group *A* and group *B* have a reasonable degree of isomorphism. In other words, we might say that no major structural differences can be expected in structures refined against data sets from group *A* and group *B*. This is illustrated by Table 7 ▶, in which group *A* (data sets 1, 5, 7, 9 and 11) has an *R*_meas_ of 0.128 (*R*_p.i.m._ = 0.048), while group *B* (data sets 2, 3, 4, 6, 8 and 10) has an *R*_meas_ of 0.119 (*R*_p.i.m._ = 0.040). When merged, their combined *R*_meas_ increases only slightly to 0.149 and *R*_p.i.m._ remains at 0.040. It can be concluded that the two groups are essentially isomorphous, with only very minor variations in structure. To support this conclusion, we refined the two structures using PDB entry 2hu1 as a starting model. The 2hu1 unit-cell parameters deposited were very close to those for groups *A* and *B*. The starting model 2hu1 was stripped of water molecules and counterions and a run of rigid-body refinement was carried out using *REFMAC*, followed by several cycles of positional refinement and individual *B*-factor refinement alternating between *REFMAC* and *Coot* for model building. TLS refinement was carried out in the last refinement cycle against group *A* data only. The results are summarized in Table 8 ▶. The two structures are essentially identical, with an r.m.s.d. using only the C^α^ atoms of 0.1 Å. The most notable differences were the number of water molecules and ions: four Cl^−^ ions, eight Na^+^ ions and 64 waters for the group *A* model and four Cl^−^ ions, seven Na^+^ ions and 57 waters in the group *B* model.

### 3.5. Cryocooled membrane protein: space group *C*2

In general, membrane-protein crystals diffract poorly. Consequently, long exposure times are required to obtain high-resolution data and often a full data set cannot be collected from a single position on the crystal owing to severe radiation damage. Generally, with large crystals data are collected at multiple positions on the crystal. Where the crystal is smaller multiple crystals must be used to generate the data. As might be expected for crystals with a high solvent content, there is a problem with non-isomorphism between different crystals and consequently many data sets are usually collected to be able to solve the structure. For this novel membrane protein, hereafter labelled memPROT, 22 data sets were collected from six crystals on the MX beamlines at Diamond Light Source. All crystals were mercury-derivatized in an attempt to provide additional phasing information. The data-set distribution across the six crystals is summarized in Table 9 ▶. Only three crystals (A34, A45 and yu60) yielded complete data sets from a single sweep at a single position on the crystal. Crystals M1S3 and M1S14 yielded 18 partial data sets, with rotation sweeps of 25° or 60°, while crystal y18 could only provide a 75% complete data set owing to radiation damage.

**Figure 8:**
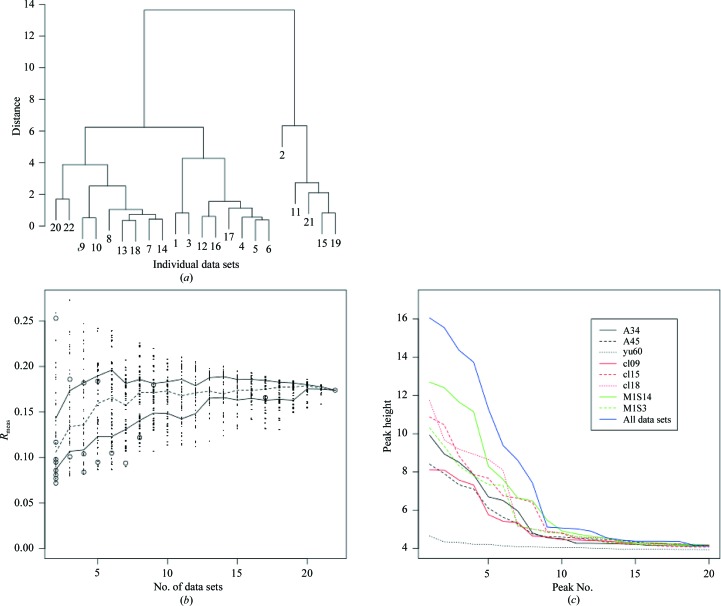
(*a>*) Dendrogram for cluster analysis of the 22 cryocooled memPROT data sets introduced in §[Sec sec3.5]3.5. (*b*) Spread of *R*_meas_ values for random combinations of 2, 3, 4, …, 21, 22 data sets (§[Sec sec3.5]3.5). The broken line joins the medians for all cases. Full lines join the inter-quartile range points for all cases. The empty circles represent *R*_meas_ for all merged data sets found in the dendrogram in (*a*). Among all data sets with an *R*_meas_ of <0.15 and a completeness of >90%, only three turn out to be useful for structure solution: those corresponding to clusters 9, 15 and 18. (*c*) Height of peaks for the strongest 20 peaks in the anomalous Fourier for the three single data sets A34, A45 and yu60, the three combined data sets corresponding to cluster 9, the two combined data sets corresponding to crystals M1S3 and M1S14, and for all data sets combined together. The anomalous maps calculated from clusters 15 and 18 clearly show higher peaks than those calculated from data set A34. The anomalous signal provided by the combined data set M1S14 (see §[Sec sec3.5]3.5) is even higher. The highest signal, as one would expect, comes from the case where all data sets are merged together, because of the good degree of isomorphism and the relatively low resolution of the data involved. The number of anomalous scatterers in the asymmetric unit (the portion of the Fourier transform shown here) is 12.

*BLEND* was used to analyse all 22 derivative data sets and produced the dendrogram in Fig. 8 ▶(*a*). The merging statistics for the combined data sets from *BLEND* are tabulated in Table 10 ▶. Random samplings of clusters with two, three, four and up to 22 data sets, as for other test cases, provide the *R*_meas_ benchmark values shown in Fig. 8 ▶(*b*) and, as in the previous examples, most of the *BLEND* merging groups show the lowest values of *R*_meas_.

Selection of the best data set from Table 10 ▶ is based on a compromise between completeness and data quality as determined by *R*_meas_ and *R*_p.i.m._. In this case merged data sets with a completeness of >90% and an *R*_meas_ of <0.15, corresponding to clusters 7, 9, 10, 15 and 18, were considered for further analysis. Of these, clusters 7 and 10 were subclusters of cluster 15 and were therefore discarded. Clusters 9, 15 and 18 were used for phasing, model building and refinement.

In order to assess the potential benefits of these three *BLEND*-selected data sets over the existing complete crystal data sets A34, A45 and yu60, anomalous difference Fourier maps (Roach, 2003 ▶) were calculated using phases from the final refined model. Peak heights for the top 20 Fourier peaks were plotted for the six data sets (Fig. 8*c* ▶). Most noticeable in Fig. 8(*c* ▶) is that the peak heights for clusters 15 and 18 are higher than those for the single-sweep data sets A34, A45 and yu60. Peak heights for cluster 9 are comparable to those for data set A45, although lower than those for data set A34. The case corresponding to the anomalous map with data from all data sets has also been added to the other curves in Fig. 8 ▶(*c*). This curve is the highest, as one would expect given the level of isomorphism and the limited resolution for this case. The use of multiple crystals/data sets is a valid alternative to single crystals whenever data from single crystals do not provide sufficient completeness and/or phasing power.

Fig. 8 ▶(*c*) also shows results for two other data sets, M1S3 and M1S14, corresponding to the combination of partial data sets collected from crystals M1S3 and M1S14, respectively. It happens that these two combined data sets are not associated with any node of the dendrogram from *BLEND*. Owing to the nature of the Ward clustering algorithm, other data sets interfered in groups belonging to either M1S3 or M1S14. This prevented, for instance, data sets 1 and 3 joining data set 2 to produce M1S3; similarly, for M1S14, clusters 15 and 12, forming most of M1S14, are prevented from merging into a single cluster because of data sets 1 and 3.

Data sets M1S3 and M1S14 were investigated in this case because they were composed of partial data sets from the same crystals. It was logical to assume that the partial data sets would be more isomorphous with each other than those from separate crystals. Thus, in this test case, looking at the favourable statistics for clusters 12 and 15 one is led to believe that perhaps all partial data sets forming crystal M1S14 could provide a complete and good quality data set. Merging statistics for M1S3 and M1S14 are appended to the bottom of Table 10 ▶. The values for M1S14 are indeed remarkably good. The anomalous signal provided by this data set is also exceedingly high compared with those of the other data sets (see full green curve in Figure 8*c* ▶).

**Figure 9:**
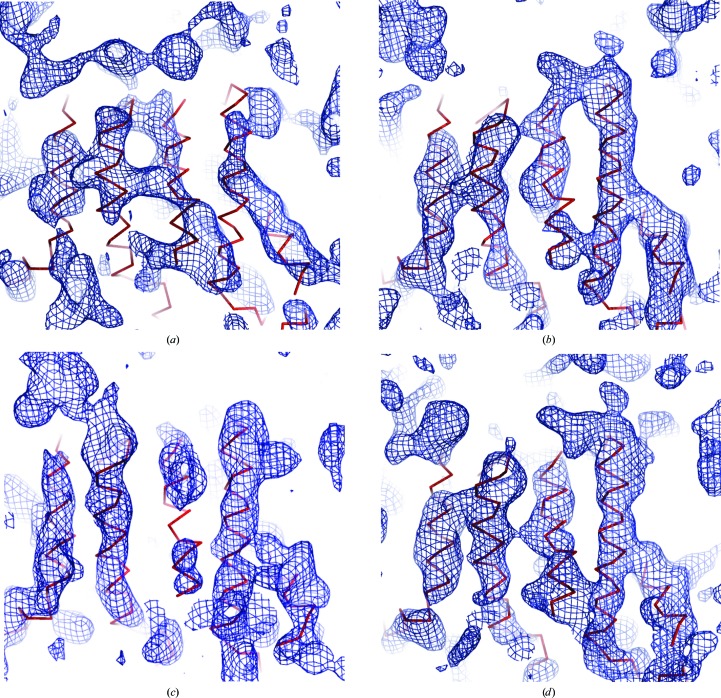
Density maps for memPROT obtained with experimental phasing in *autoSHARP* (Vonrhein *et al.*, 2007 ▶). The C^α^-backbone trace of the final refined model is overlaid in red. (*a*) A34 is the best-quality data set corresponding to a full complete sweep. (*b*) cl015 and (*c*) cl18 result from the merging of multiple data sets collected at different positions of one and two crystals, respectively. (*d*) M1S14 results from the combination of all 15 data sets collected from crystal M1S14. The increased quality for the connectivity of multiple data-set maps compared with that of the individual data set is clearly displayed.

Data set M1S14 was ultimately used to compute improved phases that resulted in electron density that showed more connectivity than that computed with other data sets. A portion of the electron density from experimental phases corresponding to different data sets is displayed in Fig. 9 ▶. The electron density calculated using A34 (Fig. 9 ▶ *a*) is visibly less connected than the electron density corresponding to M1S14 (Fig. 9 ▶ *d*). It is interesting to also observe better connectivity and more details for clusters 15 and 18 (Figs. 9 ▶ *b* and 9 ▶ *c*, respectively).

Most importantly, as cluster 18 is formed out of contributions from crystals M1S3 and M1S14, if data from M1S14 had been insufficient to form a complete data set and/or to provide any useful anomalous signal, their union with data from M1S3 would have complemented them well for phasing and model building.

This case illustrates the significant benefits of *BLEND* in providing a useful overview of numerous data sets in terms of their relative qualities and how *BLEND* can be persuasive in encouraging the combination of data from different crystals. It does, however, also identify a current weakness in *BLEND* whereby the clusters identified do not necessarily correspond to the best possible data-set combination. It is still noteworthy that *BLEND* was able to identify a cluster (cl18) comprising data from two crystals yielding a 99.1% complete data set with significant anomalous signal and resulting in a map with clearly interpretable secondary-structure features.

## 4. Conclusions and future work

In challenging (and increasingly more common) cases in which crystals are small, inhomogeneous and radiation-sensitive, complete data sets must be assembled from data from multiple crystals. A significant issue with multiple-crystal data is management and bookkeeping. *BLEND* provides a con­venient and quick way of analysing multiple data sets and presenting an informative overview of the key characteristics of the merged clusters. *BLEND* manages multiple data sets in a way that allows users not to become overburdened with tedious bookkeeping and to focus effort on obtaining high-quality, complete and redundant data.

It has been shown in several examples that, on average, clustering based on unit-cell parameters has a propensity to form data sets with the best merging statistics without the need to explore the very large space of data-set combinations.

High values of *R*_meas_ or even the failure to scale certain combinations of data sets in all test cases presented, with the exception of ultralente insulin, cannot be ascribed exclusively to non-isomorphism but also arises from data incompleteness. For instance, both cryocooled thaumatin and cryocooled insulin have an *R*_meas_ of 0.165 when merging all data sets together; this obviously means that they either come from reasonably isomorphous crystals or from reasonably isomorphous parts of the same crystal. Still, certain clusters with a small number of data sets display values of *R*_meas_ that are much higher than 0.165. This is even more the case for many random combinations of data sets. The likely reason for this to occur is the lack of sufficient overlap for partial data sets and, accordingly, the impossibility of determining sensible scaling parameters for their combination. Both good completeness and good merging statistics are important, and clustering in *BLEND* achieves exactly this. It does not sacrifice merging quality in favour of completeness, for example. *BLEND* provides an option (-c) to include data sets not present in the dendrogram. As already explained, *BLEND* has the ability to automatically select groups with good merging statistics, thus reducing the enormous and mostly unachievable task of computing all possible data-set combinations. At the same time, *BLEND* has some built-in flexibility and allows users to decide which data sets should be combined and scaled according to the desired protocol. Ultimately, *BLEND* can only deal with the available data. It is the responsibility of other software or the user to ensure that all reciprocal-space regions are covered by the data.

It would be advantageous to assess data quality on individual data sets prior to clustering. Suppose a data set presenting poor merging statistics joins a group with good statistics. For the new group the statistics will be worse because the new member of the group will have ‘polluted’ the cluster. This insertion might potentially have prevented the old group from directly joining a different data set or group of data sets that would have yielded better statistics. It is therefore very important to be able to detect bad, or rogue, data sets prior to the clustering process; the resulting dendrogram would likely appear more homogeneous. At present, no outlier rejection of this type is carried out in *BLEND*. This is principally because *BLEND* was designed to operate on very partial data sets that individually might not yield sensible merging statistics. Therefore, other quality indicators must be developed, perhaps measuring data dispersion around averages or similar statistical indicators of data concentration.

In Figs. 4 ▶(*b*) and 7 ▶(*b*) a gap is observed in the sizes of the clusters. No groups of size 6, 7 or 8 exist for the insulin ultralente case, while no groups of size 7, 8, 9 or 10 exist for *in situ* lysozyme. The reason for this is the nature of hierarchical clustering. At every node new branches are formed in the dendrogram, with each branch carrying a greater number of objects than the branches from which it originated. This process accelerates rapidly at the top of the tree, where large branches emerge out of much smaller ones. Thus, it is unlikely that clusters of specific intermediate sizes will be observed when using hierarchical clustering. Other clustering tech­niques will have to be tried in order to create groups of any number of data sets. Non-hierarchical clustering techniques, such as *k*-means clustering (MacQueen, 1967 ▶), have the ability to form clusters of all sizes, from many smallest individual objects to the single overall final cluster. Other non-hierarchical techniques, such as fuzzy clustering, can avoid the problem of forming clusters of well defined size by assigning weights that quantify the degree of association of an individual object to a given group. These clustering methods will be incorporated into *BLEND* in the future.

One of the main reasons to collect small wedges of data from multiple crystals is to avoid long crystal exposure and, accordingly, to keep intensity bias arising from radiation damage to a minimum. However, it is still desirable during the analysis of multiple data sets to try and eliminate diffraction images that appear to have been severely affected by radiation. Procedures for the robust handling of radiation damage are currently being developed.

The reduction of bias caused by radiation damage is one of the main gains derived from using multiple data sets (Hanson *et al.*, 2012 ▶) and in the extreme can be used to avoid any significant damage altogether, co-existing with other valid methods such as damage estimation, cryocooling and long exposure times in conjunction with fast readout methods (Holton & Frankel, 2010 ▶; Owen *et al.*, 2012 ▶).

An important by-product of the higher redundancy gained by collection from multiple data sets is the potential increase in anomalous signal and and resulting phasing power, as demonstrated elsewhere by Liu *et al.* (2011 ▶) and here with the memPROT example of §[Sec sec3.5]3.5. Currently, no separation of data sets based on wavelength is included in the program. Accordingly, no provisions to cater for anomalous phasing preparation are built into the code. The user will have to treat data for the same crystal at different wavelengths as different data sets and carry out cluster analysis on all of them at the same time. Enhancement of the anomalous signal is to be expected for those groups of isomorphous crystals for which data were collected at the same wavelength. *BLEND*’s management of multi-wavelength and isomorphous replacement data will be improved to assist users in the analysis of such data.

Cluster analysis, and the subsequent scaling and merging of data sets corresponding to its nodes, can be successful in grouping together isomorphous data and separating non-isomorphous data. This is, for instance, what emerges in the insulin ultralente case, where group *A* and group *B* corresponded to clear structural differences. On the other hand, in the example of *in situ* lysozyme the two main branches did not correspond to any major non-isomorphism but rather pointed to minor differences. This illustrates a level of subjectivity in drawing conclusions about whether the clusters actually reflect significant isomorphism or not. This is clearly a user-dependent choice to be made based on the level of structural detail that the user is interested in studying. Ultimately, the most informative way of using *BLEND* is to generate multiple clusters, each corresponding to different levels of similarity or isomorphism, and analyse each one up to the evaluation of electron density. In this way, *BLEND* is a valuable addition to any automated pipeline dealing with multiple data sets from multiple crystals from the same project.

## Supplementary Material

Supplementary material file. DOI: 10.1107/S0907444913012274/dz5278sup1.pdf

## Figures and Tables

**Table 1:** Linear cell variation (LCV) values for the five test cases described in §[Sec sec3]3 This quantity, introduced in §[Sec sec2.2]2.2 and detailed in Appendix *A* [App appa], is a quantitative measure of data-set isomorphism in a manner somewhat complementary to that described by dendrograms. An LCV value of 0 indicates perfect isomorphism. The larger the value of LCV, the higher the non-isomorphism among the data sets being considered.

Test case	LCV (%)
Cryo-thaumatin	1.1
Cryo-insulin	1.0
Ultralente	2.0
*In situ* lysozyme	0.7
MemPROT	1.4

**Table 2:** Merging statistics for the optimal merging combinations suggested by *BLEND* for the cryocooled thaumatin data sets

Data sets	*R*_meas_	*R*_p.i.m._	Completeness (%)	Multiplicity	〈*I*/σ(*I*)〉	Lowest resolution (Å)	Highest resolution (Å)
6, 7	0.037	0.016	63.8	4.9	37.9	40.84	1.95
5, 11	0.052	0.030	77.2	2.6	17.8	40.84	1.95
6, 7, 5, 11	0.040	0.014	77.2	6.6	39.0	40.84	1.95
8, 10	0.072	0.047	72.7	1.7	13.9	37.49	1.95
2, 4	0.092	0.056	59.3	2.7	48.8	35.78	1.95
13, 14	0.091	0.059	61.1	1.7	14.4	57.28	1.95
3, 2, 4	0.089	0.043	60.1	4.0	38.4	35.78	1.95
9, 8, 10	0.097	0.051	91.1	3.1	21.5	40.90	1.95
6, 7, 5, 11, 9, 8, 10	0.115	0.038	95.9	8.1	31.3	40.86	1.95
1, 6, 7, 5, 11, 9, 8, 10	0.132	0.044	98.8	8.6	32.3	40.85	1.95
12, 3, 2, 4	0.096	0.045	78.6	3.7	25.8	49.94	1.95
13, 14, 12, 3, 2, 4	0.160	0.070	93.2	4.2	27.6	57.46	1.95
All data sets	0.165	0.047	99.9	12.4	32.2	57.64	1.95

**Table 3:** Merging statistics for the merging sets suggested by *BLEND* for the cryocooled insulin data

Data sets	*R*_meas_	*R*_p.i.m._	Completeness (%)	Multiplicity	〈*I*/σ(*I*)〉	Lowest resolution (Å)	Highest resolution (Å)
13, 14	0.069	0.047	81.1	1.5	14.2	24.32	1.92
8, 11	0.105	0.063	68.9	1.8	27.3	30.19	1.92
1, 7	0.112	0.076	77.6	1.5	16.6	23.58	1.92
3, 13, 14	0.082	0.049	85.0	2.2	17.4	30.29	1.92
1, 6, 7	0.278	0.175	91.0	1.9	15.6	24.36	1.92
10, 12	0.104	0.062	61.7	2.0	9.1	30.17	1.92
1, 4, 6, 7	0.166	0.114	59.5	1.3	45.9	24.36	1.92
8, 10, 11, 12	0.109	0.054	84.8	2.9	14.6	30.18	1.92
2, 3, 13, 14	0.099	0.058	93.5	2.5	16.2	30.30	1.92
1, 4, 5, 6, 7	0.203	0.112	97.7	3.0	41.4	30.35	1.92
8, 9, 10, 11, 12	0.138	0.073	94.2	3.1	13.6	30.18	1.92
1, 2, 3, 4, 5, 6, 7, 13, 14	0.150	0.064	99.7	5.3	22.6	30.33	1.92
All data sets	0.165	0.057	99.9	8.2	25.0	30.27	1.92

**Table 4:** Results for the ultralente insulin data set used to solve the structure, reproduced from Wagner *et al.* (2009 ▶) The top part of this table displays values from data integration and data scaling; the bottom part relates to values after structure refinement. Values in parentheses are for the high-resolution data range.

Beam transmission (%)	30
Wavelength (Å)	0.9786
Unit-cell parameters (Å)	*a* = 81.03, *c* = 33.90
Resolution range (Å)	41.0–2.2 (2.3–2.2)
〈*I*/σ(*I*)〉	11.8 (5.7)
Completeness (%)	98.8 (98.7)
*R*_merge_ (%)	7.2 (20.9)
Resolution range (Å)	40.5–2.2
*R*/*R*_free_ (%)	24.0/28.2
PDB code	2vk0

**Table 5:** Merging statistics for the clusters suggested by *BLEND* for the ultralente insulin test case

Data sets	*R*_meas_	*R*_p.i.m._	Completeness (%)	Multiplicity (%)	〈*I*/σ(*I*)〉	Lowest resolution (Å)	Highest resolution (Å)
6, 8	0.129	0.069	80.6	2.8	6.4	23.46	1.83
1, 4	0.172	0.106	88.3	1.9	3.3	24.38	1.83
3, 1, 4	0.196	0.114	91.5	2.5	4.3	24.39	1.83
5, 3, 1, 4	0.194	0.104	98.5	3.0	4.4	24.40	1.83
7, 9	0.190	0.122	87.5	1.7	4.0	24.25	1.83
2, 5, 3, 1, 4	0.199	0.100	99.5	3.7	5.0	24.37	1.83
6, 8, 7, 9	0.223	0.110	98.4	3.8	5.6	24.32	1.83
All data sets	0.406	0.145	99.7	7.4	5.1	24.35	1.83

**Table 6:** Final statistics for structures related to group *A* and group *B* of ultralente insulin The values in parentheses are for the high-resolution data range.

	Group *A*	Group *B*
Resolution range (Å)	24.36–2.10 (2.21–2.10)	24.32–2.00 (2.11–2.00)
*R*_merge_	0.138 (0.799)	0.185 (0.584)
*R*_meas_	0.153 (0.888)	0.203 (0.658)
*R*_p.i.m._	0.065 (0.382)	0.085 (0.289)
〈*I*/σ(*I*)〉	7.4 (2.3)	7.4 (2.8)
Multiplicity	5.1 (5.1)	5.1 (5.1)
Completeness (%)	99.9 (100.0)	99.5 (100.0)
No. of reflections	24303	28373
No. of unique reflections	4775	5551
*R*_work_/*R*_free_ (%)	18.5/24.8	20.9/28.0
R.m.s.d., bonds (Å)	0.016	0.015
R.m.s.d., angles (°)	1.923	1.877
Unit-cell parameters (Å)		
*a*	80.05	81.02
*c*	34.25	33.75

**Table 7:** Merging statistics for the clusters suggested by *BLEND* for the *in situ* lysozyme test case

Data sets	*R*_meas_	*R*_p.i.m._	Completeness (%)	Multiplicity	〈*I*/σ(*I*)〉	Lowest resolution (Å)	Highest resolution (Å)
7, 11	0.092	0.052	84.1	2.6	9.4	39.38	1.86
2, 3	0.091	0.048	78.0	2.6	9.5	35.31	1.86
5, 7, 11	0.112	0.054	89.7	3.6	10.1	39.39	1.86
4, 6	0.095	0.053	75.8	2.3	9.6	35.38	1.86
8, 10	0.110	0.046	50.2	4.3	19.0	39.54	1.86
9, 5, 7, 11	0.112	0.048	92.9	4.6	11.2	39.38	1.86
2, 3, 4, 6	0.115	0.050	93.2	4.0	9.8	35.34	1.86
1, 5, 7, 9, 11	0.128	0.048	93.3	5.8	11.9	39.37	1.86
2, 3, 4, 6, 8, 10	0.119	0.040	94.5	6.3	10.7	39.52	1.86
All data sets	0.149	0.040	98.5	11.5	15.2	39.45	1.86

**Table 8:** Final statistics for structures related to group *A* and group *B* of *in situ* lysozyme Values in parentheses are for the high-resolution data range.

	Group *A*	Group *B*
Resolution range (Å)	39.37–1.86 (1.96–1.86)	39.52–1.86 (1.96–1.86)
*R*_merge_	0.117 (0.267)	0.111 (0.313)
*R*_meas_	0.128 (0.320)	0.119 (0.373)
*R*_p.i.m._	0.048 (0.172)	0.040 (0.197)
〈*I*/σ(*I*)〉	10.8 (3.3)	10.9 (2.7)
Multiplicity	5.8 (2.7)	6.3 (2.7)
Completeness (%)	93.3 (82.2)	94.5 (86.7)
No. of reflections	56832	63513
No. of unique reflections	9831	10070
*R*_work_/*R*_free_ (%)	16.0/21.1	15.3/20.2
R.m.s.d., bonds (Å)	0.019	0.018
R.m.s.d., angles (°)	1.882	1.881
Unit-cell parameters (Å)		
*a*	78.73	79.05
*c*	38.50	38.51

**Table 9:** Data sets collected for the memPROT structure Multiple data sets were obtained from the first two crystals, M1S3 and M1S14, by measuring at several different locations on the same crystal.

Crystal name	Total No. of data sets	Serial data set Nos.	Combined data set name
M1S3	3	1, 2, 3	M1S3
M1S14	15	4, 5, 6, 7, 8, 9, 10, 11, 12, 13, 14, 15, 16, 17, 18	M1S14
A34	1	19	A34
A45	1	20	A45
y18	1	21	y18
yu60	1	22	yu60

**Table 10:** Merging statistics for the selection suggested by cluster analysis The data sets forming these selections relate to memPROT, introduced in §[Sec sec3.5]3.5. The combined data sets M1S3 and M1S14 are not associated with any node in the dendrogram of Fig. 8 ▶, but they correspond to the individual crystals M1S3 and M1S14, respectively.

Cluster	Data sets	*R*_meas_	*R*_p.i.m._	Completeness (%)	Multiplicity	〈*I*/σ(*I*)〉	CC_anom_	Lowest resolution (Å)	Highest resolution (Å)
1	13, 18	0.072	0.051	75.60	1.3	5.8	0.000	53.93	4.21
2	5, 6	0.098	0.069	67.70	1.5	5.2	0.000	61.73	4.21
3	7, 14	0.086	0.045	28.80	3.4	11.1	0.258	53.25	4.21
4	9, 10	0.081	0.057	66.20	1.5	6.0	0.000	61.50	4.21
5	4, 5, 6	0.101	0.070	82.80	1.8	6.5	0.720	61.73	4.21
6	12, 16	0.077	0.048	56.60	1.8	6.7	0.178	61.61	4.21
7	13, 18, 7, 14	0.084	0.047	90.70	2.2	11.3	0.273	53.92	4.21
8	1, 3	0.095	0.052	75.20	3.0	12.1	0.302	54.16	4.21
9	15, 19	0.117	0.066	98.70	2.7	7.1	−0.165	54.16	4.21
10	8, 13, 18, 7, 14	0.095	0.046	91.10	2.7	7.9	0.232	53.93	4.21
11	17, 4, 5, 6	0.104	0.062	83.10	2.4	7.7	0.141	61.74	4.21
12	12, 16, 17, 4, 5, 6	0.105	0.053	84.00	3.5	10.2	0.089	61.70	4.21
13	20, 22	0.253	0.126	99.40	4.0	9.5	−0.220	112.56	4.21
14	21, 15, 19	0.186	0.089	99.40	4.0	8.8	−0.402	66.80	4.21
15	9, 10, 8, 13, 18, 7, 14	0.094	0.047	93.90	3.7	9.4	0.167	61.56	4.21
16	11, 21, 15, 19	0.182	0.084	99.70	4.5	6.8	−0.380	66.81	4.21
17	20, 22, 9, 10, 8, 13, 18, 7, 14	0.180	0.067	99.90	7.4	8.0	0.034	112.53	4.21
18	1, 3, 12, 16, 17, 4, 5, 6	0.122	0.051	99.10	5.3	10.7	0.087	61.79	4.21
19	20, 22, 9, 10, 8, 13, 18, 7, 14, 1, 3, 12, 16, 17, 4, 5, 6	0.166	0.047	99.90	12.6	18.9	0.146	112.62	4.21
20	2, 11, 21, 15, 19	0.184	0.079	99.70	5.3	7.9	−0.275	66.83	4.21
21	All data sets	0.174	0.042	99.90	18.0	16.8	0.136	112.80	4.21
M1S3	1, 2, 3	0.099	0.053	95.50	3.2	23.2	0.261	54.28	4.21
M1S14	4, 5, 6, 7, 8, 9, 10, 11, 12, 13, 14, 15, 16, 17, 18	0.098	0.036	98.90	7.5	11.4	0.275	61.67	4.21

## Footnotes

1 Supplementary material has been deposited in the IUCr electronic archive (Reference: DZ5278). Services for accessing this material are described at the back of the journal.

## Appendix A The linear cell variation

A unit cell is characterized by six parameters: the three side lengths *a*, *b* and *c* and the three angles α, β and γ. A structural change is normally followed by changes both in side lengths and angles. These can be described simply as changes in length if we consider the diagonals across three independent faces of the unit cell. If we indicate with *D*_*ab*_ the diagonal length on the face defined by vectors **a** and **b**, and use similar notation for the other two diagonals, we have

Now, as the linear cell variation is meant to provide the extent of change as arising from non-isomorphism, it should measure the largest variation across the three diagonals. Furthermore, we would like to measure such variation in fractional rather than absolute terms. Thus, we build three square matrices, *M*_*ab*_, *M*_*ac*_, *M*_*bc*_, the elements of which are the relative differences between diagonals,

where, for instance, *D_ab_*(*i*) and *D_ab_*(*j*) are the values of diagonal *D_ab_* for data sets *i* and *j*, respectively. The largest element of these matrices,

is the linear cell variation defined in §[Sec sec2.2]2.2.
