# Supplementary material for: Clustering procedures for the optimal selection of data sets from multiple crystals in macromolecular crystallography
Source: Acta Crystallogr D Biol Crystallogr. 2013 Jul 20;69(Pt 8):1617–32. doi: 10.1107/S0907444913012274 (PMC3727331; doi:10.1107/S0907444913012274)
Supplement: Supplementary file 1 [file d-69-01617-sup1.pdf]

Supplementary Material

Clustering procedures for the optimal selection of datasets from multiple crystals in macromolecular crystallography

The following tables include information related to data collection for the five test cases described in the main text.

Dataset	Angular range (degrees)	Angular step (degrees)	Number of images	Crystal size (μm)	a (Å)	c (Å)	Highest resolution (Å)
1	10	0.250	40	500 × 500	57.648	150.251	1.501
2	10	0.250	40	200 × 200	57.605	149.847	1.147
3	10	0.250	40	200 × 200	57.588	149.705	1.906
4	10	0.250	40	200 × 200	57.590	149.808	1.147
5	20	0.500	40	100 × 200	57.758	150.071	1.294
6	20	0.500	40	100 × 200	57.754	150.060	1.296
7	20	0.500	40	100 × 200	57.752	150.057	1.299
8	8	0.200	40	N/A	57.832	149.975	1.091
9	20	0.500	40	100 × 100	57.874	150.090	1.094
10	8	0.200	40	200 × 200	57.798	149.969	1.318
11	6	0.150	40	100 × 100	57.764	150.060	1.537
12	8	0.200	40	N/A	57.364	149.911	1.536
13	8	0.200	40	50 × 40	57.307	150.205	1.539
14	8	0.200	40	30 × 30	57.258	150.320	1.663

Table 1: Data collection parameters and unit cell dimensions for the cryo-cooled thaumatin test data sets. All crystal sizes are approximate and where N/A is given no crystal size was recorded.

Dataset	Angular range (degrees)	Angular step (degrees)	Number of images	a (Å)	c (Å)	Highest resolution (Å)
1	20	1.000	20	81.660	33.610	1.759
2	20	1.000	20	81.410	33.570	1.759
3	20	1.000	20	81.550	33.550	1.759
4	20	1.000	20	81.590	33.630	1.759
5	20	1.000	20	81.900	33.570	1.759
6	20	1.000	20	81.780	33.590	1.759
7	20	1.000	20	81.700	33.580	1.759
8	20	1.000	20	81.450	33.420	1.759
9	20	1.000	20	81.060	33.420	1.759
10	20	1.000	20	81.300	33.340	1.759
11	20	1.000	20	81.410	33.390	1.759
12	20	1.000	20	81.310	33.430	1.759
13	20	1.000	20	81.630	33.520	1.759
14	20	1.000	20	81.610	33.510	1.759

Table 2: Data collection parameters for the cryo-cooled insulin test data.

Dataset	Angular range (degrees)	Angular step (degrees)	Number of images	a (Å)	c (Å)	Highest resolution (Å)
1	40	1.000	40	80.100	34.250	1.784
2	40	1.000	40	79.730	34.040	1.780
3	30	1.000	30	80.160	34.340	1.781
4	30	1.000	30	80.050	34.310	1.783
5	30	1.000	30	80.290	34.400	1.781
6	60	1.000	60	81.270	33.750	1.780
7	40	1.000	40	80.480	33.680	1.780
8	40	1.000	40	81.290	33.790	1.674
9	30	1.000	30	80.870	33.800	1.676

Table 3: Details for datasets of cryo-cooled ultralente.

Dataset	Angular range (degrees)	Angular step (degrees)	Number of images	a (Å)	c (Å)	Highest resolution (Å)
1	20	0.200	100	78.595	38.664	1.744
2	20	0.200	100	78.961	38.392	1.741
3	20	0.200	100	78.940	38.455	1.776
4	20	0.200	100	79.051	38.431	1.729
5	20	0.200	100	78.826	38.436	1.727
6	20	0.200	100	79.162	38.461	1.737
7	20	0.200	100	78.750	38.480	1.728
8	20	0.200	100	79.161	38.600	1.862
9	20	0.200	100	78.706	38.355	1.734
10	20	0.200	100	78.989	38.695	1.739
11	20	0.200	100	78.784	38.517	1.861

Table 4: Details for datasets of lysozyme crystals, recently collected *in situ*, at room temperature, on I04.1 beam line at the Diamond Light Source synchrotron.

Crystal	Dataset	Angular range (degrees)	Angular step (degrees)	Number of images	a (Å)	b (Å)	c (Å)	β (degrees)	Highest resolution (Å)
M1S3	1	60	0.500	120	152.31	172.37	272.13	100.94	3.406
M1S3	2	60	0.500	120	153.50	172.30	271.95	100.36	3.406
M1S3	3	60	0.500	120	152.48	172.36	271.72	100.96	3.406
M1S14	4	25	0.500	50	152.49	171.48	272.10	101.29	3.867
M1S14	5	25	0.500	50	152.37	171.40	272.30	101.23	3.867
M1S14	6	25	0.500	50	152.30	171.40	272.10	101.26	3.867
M1S14	7	25	0.500	50	152.20	170.81	271.30	101.23	3.867
M1S14	8	25	0.500	50	152.24	171.49	271.62	101.25	3.867
M1S14	9	25	0.500	50	152.13	170.90	270.81	101.26	3.867
M1S14	10	25	0.500	50	152.19	170.94	270.83	101.13	3.867
M1S14	11	25	0.500	50	152.74	171.82	273.35	101.59	3.867
M1S14	12	25	0.500	50	152.41	171.08	272.00	101.23	3.867
M1S14	13	25	0.500	50	152.24	171.12	271.55	101.25	3.867
M1S14	14	25	0.500	50	152.20	170.82	271.56	101.21	3.867
M1S14	15	25	0.500	50	152.87	172.79	272.77	101.20	3.867
M1S14	16	25	0.500	50	152.30	170.75	271.92	101.29	3.867
M1S14	17	25	0.500	50	152.56	171.63	272.10	101.08	3.867
M1S14	18	25	0.500	50	152.36	171.05	271.48	101.23	3.867
A34	19	110	0.500	220	152.77	173.17	272.60	101.33	3.378
A45	20	180	0.500	360	151.77	171.35	271.45	101.59	3.929
yu18	21	100	0.500	200	153.24	172.41	272.47	101.53	3.378
yu60	22	102	0.200	510	152.29	171.92	271.82	101.58	2.956

Table 5: Details for datasets of cryo-cooled memPROT collected at Diamond Light Source synchrotron phase-I beam lines in 2010.
